# Enhancing emergency care in low-income countries using mobile technology-based training tools

**DOI:** 10.1136/archdischild-2016-310875

**Published:** 2016-09-22

**Authors:** Hilary Edgcombe, Chris Paton, Mike English

**Affiliations:** 1 Nuffield Department of Anaesthetics, Oxford University Hospitals NHS Trust, Oxford, UK; 2 Nuffield Department of Medicine, Centre for Tropical Medicine and Global Health, University of Oxford, Oxford, UK; 3 KEMRI-Wellcome Trust Research Programme, Nairobi, Kenya

**Keywords:** Medical Education, Technology, Tropical Paediatrics

## Abstract

In this paper, we discuss the role of mobile technology in developing training tools for health workers, with particular reference to low-income countries (LICs). The global and technological context is outlined, followed by a summary of approaches to using and evaluating mobile technology for learning in healthcare. Finally, recommendations are made for those developing and using such tools, based on current literature and the authors' involvement in the field.

## The context

The global need for more and better-trained health workers, particularly in low-resourced regions, is well established.^1^ Effective training enhances the knowledge and skills of those already working and supports and motivates those who consider joining the healthcare workforce.^2^ However, delivery of timely, effective and up-to-date teaching is challenging in many low-income countries (LICs), for reasons which may be financial, geographical, political and/or institutional.^2^ Global child health is no exception to this rule.^3^ ^4^

The demand for better, more accessible training is thus considerable and over recent decades many solutions have been proposed and implemented, some originating within local/national institutions, others involving external (often high-income country (HIC)) partnerships and initiatives (eg, the Rwanda Human Resources for Health Program.^5^) The scope and nature of these solutions vary widely from long-term international partnerships to training delivered by regional associations to one-off short courses taught by volunteers. It seems likely that in LICs, as in HICs, a mix of approaches is required.

Two aspects of this training challenge warrant particular emphasis. The first is the need for contextually appropriate training which has relevance to the disease burden and resources encountered by health professionals in their workplaces. Examples of such training exist already in a variety of formats (such as “Emergency Triage Assessment and Treatment plus admission care”^6^ ^7^ and the Helping Babies Breathe collaboration).^8^ The second aspect is that of scale: even within the depleted current workforce, tens of thousands of health workers need to access initial and continuing medical education. This challenge is considerable even in well-resourced countries such as the UK: as an illustration, despite recognition that knowledge decay occurs in a matter of months after resuscitation training,^9^ it is deemed impractical to require recertification more often than once every 3–4 years. Developing tools and systems which can provide health workers in even the remotest regions with up-to-date information and timely training must be a priority. Initiatives which support self-driven learning using electronic resources, such as the Bettercare system,^10^ seem likely to become increasingly accessible and will likely be supported by the burgeoning availability of smart mobile technology.

In Africa, mobile phone use is now commonplace: in Kenya, for example, over 80% of the population now have access to a mobile phone (increased from only 10% in 2002).^11^ Farmers are using text message services to price their goods and citizens have quickly adopted the mPesa mobile payments system. It is often said that the mobile phone network in Africa has ‘leapfrogged’ the landline network; changes which took decades of investment in HICs have been overtaken within a few short years in Africa. Health initiatives using voice and text features such as appointment reminders^9^ ^12^ and treatment adherence prompts^10^ ^13^ are thus increasing in number and scope. For professional health workers who might be most interested in mobile-based learning, smartphone adoption has accelerated sharply in the past 2–3 years. Already 28% of people in Kenya with secondary-level or higher education now own a smartphone,^11^ as do 88% of Nairobi medical students.^14^ Many of these smartphone owners may never have used a laptop or desktop computer but are proficient application (app) users. It could be argued that new interventions should therefore adopt a ‘mobile-first’ approach instead of first designing for personal computers with a mobile ‘option’.

Recognising that mobile devices and internet connections bring their own strengths and weaknesses to the field of healthcare education, we examine what has been established so far about ‘mobile learning in healthcare’ which is of relevance to the low-resource setting. For the purposes of this article, we focus on mobile devices providing touchscreen technology and internet connection via phone (2G, 3G or 4G) or wireless networks.

## Mobile technology and learning in healthcare

### Models of learning using mobile technology

The challenge with any new tool, for training or otherwise, is to establish where it is of most benefit (and where it is not). The aims of training and learning in healthcare include information accessibility and transfer and encompass a far wider range of cognitive, motor, attitudinal and other end points. Mobile devices have some unique features which may assist with information transfer and knowledge retention and may assist with some of the wider aims of training in healthcare. As an example, a particular challenge in the emergency clinical setting is the ability to correctly sequence a complex series of actions appropriate to situational cues in real time, as in the resuscitation algorithm. Opportunities to practise such sequences of fast, accurate cue-response activity can be relatively rare in the clinical setting and expensive using high-fidelity simulator technology; mobile devices could enable rehearsal and assessment of both speed and accuracy of algorithm recall, easily repeated to reinforce learning. While such ideas are of considerable interest, it is important at the same time to acknowledge that there are aspects of training which do not lend themselves to mobile-assisted techniques: for example, learning motor skills is unfeasible without (currently) expensive haptic feedback technology. Communication (verbal and non-verbal) and team-working skills are similarly not (yet) readily taught using these devices. Such limitations and the little that is currently known about the educational effectiveness of mobile technology in learning mandate careful evaluation and development approaches, which are further discussed below.

Several healthcare training apps have been developed to date and the approaches used can broadly be divided into two categories. Some simply replicate existing teaching strategies ‘on a screen’, for example, by providing questions and answers for exam practice or displaying textbook graphics. Others take advantage of features specific to mobile devices, examples of which include the ability to respond with different pathways to user choices, the use of animations with which the user can interact and accelerometry (the detection of orientation and movement of a device). Table 1 demonstrates the various types of training tools which have been developed for medical training using mobile technology, with examples from the recent literature.

**Table 1:** Examples of existing mobile training tools

Training mechanics	Examples in healthcare education
Presentation of existing static teaching resources (documents, algorithms, illustrations)	Application (app) developed to enable easy access to a bioinformatics dictionary for clinicians working in clinical genomics.^15^
Presentation of videos, animations and podcasts (visual±audio components)	Recording and presenting gross pathology examination videos to residents.^16^
Communication between trainer and trainee (evaluations, supervision, reflection)	Use of QR codes combined with electronic surveys to complete residents' evaluations.^17^ ‘Telepresent supervision’ using smartphone with FaceTime connection for medical students learning tracheal intubation.^18^ App to prompt and enable immediate reflection in the workplace for residents.^19^ Use of a smartphone app to complement web-based evaluations by students of their placements.^20^
Augmented and/or virtual reality	Use of a ‘virtual’ airway visualised on a mobile phone combined with accelerometry to train medical students in fibreoptic airway techniques.^21^
Quizzes/test questions	Delivery of two questions a day to general surgical residents via a dedicated app (UF surgery), with notification and reminders to complete the questions. Immediate feedback is provided.^22^
Detection of movement, sound and other parameters	Illustration of the effects of different exercise types on human physiology: medical and biology students undertake exercise and use apps to measure their own heart rate, reaction times, respiratory rate, movement and vasodilation.^23^ CPR apps (several) provide real-time feedback on rate and depth of cardiac compressions, eg RCP Coach.^24^
Social media use	Use of dedicated Twitter account to relay factual knowledge to medical students.^25^
Anonymised interactions (surveys, in-lecture polling and feedback)	Comparison of ‘clickers’ with smartphones for student interaction in lectures.^26^
Games	Game based on serial decision trees and virtual patients to teach antimicrobial stewardship.^27^ Three-dimensional environment game for advanced life support re-training.^28^

One area of particular current interest is that of ‘games for learning’ (also called ‘serious games’, ‘educational games’ or ‘persuasive games’ among other terms). The concept of a ‘game’ may include features which encourage competition (and repetition) such as the ability to win, beat a previous score, attain badges or rewards of some kind and compete with others. Alternatively (or additionally), it may involve broader concepts of gameplay, including discovery, exploration, fun and ‘safe failure’.^29^ ^30^ Given the success and addictiveness of some mobile games which were not designed with a primary educational purpose, the question arises whether some or all these features could be usefully adapted for training. Opinion is divided on the best way to evaluate training using these tools and the majority of attempts to date have focused on the subjective and immediate experience of the player, rather than longer-term educational outcomes. The evidence, so far, is limited but positive.^31^ ^32^

### Evaluating and assessing mobile technology in healthcare learning

Critical evaluation in this field is only at an early stage and the majority of published work so far which evaluates mobile learning tools has originated from HICs. This is unsurprising, given both the ubiquity of mobile devices in the high-income world and the relative scarcity of research in the field of medical education in low/middle-income countries (LMICs). Depending on the outcomes of interest, a wide variety of approaches are employed to demonstrate user satisfaction (surveys, interviews), knowledge improvement and/or retention (pre-intervention and post-intervention testing), markers of improved performance (either in the intervention itself or a ‘gold standard’ equivalent such as simulation), data available from analytics built into the app (providing information on user uptake, completion, speeds, etc), attitudes towards the training tool (focused interviewing) and cost-benefit analysis. The challenges of evaluating technology-enhanced learning (including but not limited to mobile technologies) have been helpfully addressed in a recent review,^33^ which also makes useful recommendations for high-quality evaluation.

The key considerations may be summarised as follows: first, as detailed above, the need to develop evaluation strategies for mobile technology which produce robust information about a training tool's effectiveness. Second, the relevance of tools designed for high-income settings to very different resource-poor environments must be assessed, accepting that what works in one place may not in another (for reasons which may be related as much to differences in learning culture as to differences in clinical context).^34^ Third, there is great opportunity to analyse and evaluate ways in which mobile technology may be blended with other training modalities (such as face-to-face training) to maximise benefit and ultimately to improve healthcare outcomes.

## Suggested approaches

As a team working to develop mobile technology-assisted training in the field of paediatric emergency care in LMICs and taking into account the situation outlined above, we suggest that the following principles may be useful both for other developers and for those considering using such tools in their professional lives.

### Principle 1: adopt a development strategy appropriate for the rapidly moving world of mobile technology

The development challenge is to determine principles of design and use for new software while anticipating and planning for considerable changes in technology within a short space of time. Characteristically, software apps for mobile devices such as tablets and smartphones are developed so as to take advantage of their ability to rapidly reach a large user-base through ‘app stores’ and to gather data about how people use the software. This process has been termed the ‘Lean Startup’ by author and app developer Eric Ries and it incorporates the Agile software development principles with a process of ‘customer development’ (discovering and validating a market for the product). Following this process, developers usually release an app at a ‘minimal viable product’ stage, then aim to continuously improve the app through rapid cycles, building new versions of the product and measuring changes in usage, to learn whether the new version is better than the previous one. These cycles, called ‘build-measure-learn’ loops, are often performed using a process of randomisation whereby different users are randomly assigned different versions of the app to test different functionality. These tests can number in the millions of users for some of the most popular commercial apps. Such an approach of rapid iterative development (which shares many characteristics with the ‘design-based research’ paradigm)^35^ enables responsiveness to changes in technology and persistent improvement in the app. This process may be combined with or ultimately lead on to subsequent evaluation using more familiar qualitative and quantitative study designs.

### Principle 2: partnership and collaboration between high-income and low-income settings

While it may seem obvious, training tools are likely to need modification to be appropriate for different environments. Mobile platforms offer an opportunity for ongoing updates and changes with relative ease while maintaining a high-quality learning framework, as content can be updated remotely. In order to maximise accessibility, relevance and validity for the learner in a particular setting, those from that setting must be involved in the development process, either primarily or in partnerships. Missing this opportunity is likely to impair the designers' ability to develop the most useful software, to achieve uptake and usage and may well lead to substandard outcomes.

In our practice, for example, to ensure stakeholders are included in the design and development process, we have run co-design workshops with potential end-users (Kenyan healthcare workers) in the UK and Kenya. We have used the ‘Lean UX’ methodology, in which groups of stakeholders from both countries work together to develop user personas, value propositions (identifying solutions to key ‘pain-points’ for the user), co-design wireframes and metrics, ultimately leading to the development of a minimal viable product which can be tested using the iterative process described above. Such an approach has increasing traction internationally. Additionally, start-up incubators (such as the iHub in Nairobi, Kenya)^36^ may enable linkage to local resources and knowledge locally helping such projects to be increasingly driven by LMIC developers and designers.

### Principle 3: ambition to evaluate effectiveness in the medium-to-long term

In the context of mobile technology, two aspects of evaluation are possible. First (as for any training intervention), the assessment of the training tool against educational outcomes and/or clinical outcomes: what ‘educational success’ looks like determines the nature of this evaluation.^33^ Second, however, mobile technology lends itself to a host of other analytic tools: uptake (downloads), completion, repetition, scores in training and so forth. If considered and incorporated at the design stage, these analytics can reveal useful information about the ways in which learners use training tools which in turn can inform development.

### Principle 4: recognition of the limitations and qualifications of mobile technology

As outlined previously, not all relevant clinical skills can be taught using a mobile device. This is obvious, but important to remember amidst the hype and enthusiasm for new technological solutions. It is possible that overconfidence may be a result of training using these tools, unless the learner is clear about what they can and cannot learn effectively. Consequently, any evaluation of such training tools should allow for the detection of possible harms.

## Conclusion

We recognise and emphasise the ongoing need for training of healthcare workers in LMICs. While workforce training is only part of the global health story, it is crucial to improve health outcomes. The worldwide shift to mobile technology, which is occurring rapidly in both LMIC and HIC settings, offers the opportunity to explore mobile-based training apps as potential tools with which to improve access to training for health workers worldwide. As yet little evidence exists on how to do this most effectively, and what success might look like, and so we urge developers and clinicians to produce training tools and evaluate them rigorously, in partnership with learners, in order to maximise their effectiveness and improve global health.
